# Genebank Phenomics: A Strategic Approach to Enhance Value and Utilization of Crop Germplasm

**DOI:** 10.3390/plants9070817

**Published:** 2020-06-29

**Authors:** Giao N. Nguyen, Sally L. Norton

**Affiliations:** Australian Grains Genebank, Agriculture Victoria, 110 Natimuk Road, Horsham 3400, Australia; sally.norton@agriculture.vic.gov.au

**Keywords:** high-throughput phenotyping, statistical modelling, phenotypic breeding, genomic selection

## Abstract

Genetically diverse plant germplasm stored in ex-situ genebanks are excellent resources for breeding new high yielding and sustainable crop varieties to ensure future food security. Novel alleles have been discovered through routine genebank activities such as seed regeneration and characterization, with subsequent utilization providing significant genetic gains and improvements for the selection of favorable traits, including yield, biotic, and abiotic resistance. Although some genebanks have implemented cost-effective genotyping technologies through advances in DNA technology, the adoption of modern phenotyping is lagging. The introduction of advanced phenotyping technologies in recent decades has provided genebank scientists with time and cost-effective screening tools to obtain valuable phenotypic data for more traits on large germplasm collections during routine activities. The utilization of these phenotyping tools, coupled with high-throughput genotyping, will accelerate the use of genetic resources and fast-track the development of more resilient food crops for the future. In this review, we highlight current digital phenotyping methods that can capture traits during annual seed regeneration to enrich genebank phenotypic datasets. Next, we describe strategies for the collection and use of phenotypic data of specific traits for downstream research using high-throughput phenotyping technology. Finally, we examine the challenges and future perspectives of genebank phenomics.

## 1. Introduction

The global population is forecasted to reach 9.6 billion people by 2050, with the current 1.3% annual growth rate of crop productivity required to increase to 2.4% to meet the expected food security needs [1]. Concomitantly, climate change is impacting global food and biofuel production chains through rising temperatures, increasing carbon dioxide concentrations, unpredictable rainfall patterns, and soil degradation [2]. Extreme weather conditions, such as drought and heat that occurs during critical crop growth phases, reduce yield and production of major grain crops, and can trigger the emergence of new pests and diseases that can cause further production losses [2,3]. To produce enough food to meet this increasing demand under current and future growing conditions, it is critical that plant breeders develop new high yielding, environmentally resilient, and sustainable crop varieties.

Crop improvement through breeding relies heavily on genebanks worldwide to provide genetically diverse material that contains genes and alleles that govern desirable agronomic traits [4]. Novel alleles discovered from genebank genetic resources underpin the selection of, and enhance the genetic gain in breeding programs for favorable traits such as high yield, abiotic, and biotic stress adaptation [5,6,7]. However, under current practices, most genebanks can only offer end-users limited passport and basic characterization data based on morphological traits guided by standard international descriptors [8]. Only a small number of accessions have agronomic and quality trait data available [9]. There are around 1750 individual genebanks worldwide that preserve approximately 7.4 million accessions of agricultural genetic materials [10]. However, only 10% of these accessions are used for breeding purposes, partly due to poor phenotypic and genotypic characterization or lack of evaluation for agronomic traits [11] or because they are not publicly available [5].

Li et al. [12] pointed out two major limitations preventing the exploitation of genebank genetic resources for breeding programs: 1) time and available resources for thorough characterization of accessions at a large scale; and 2) identifying and introducing the allelic variance into elite breeding materials. This missing characterization data makes searching for an accession with specific desirable agronomic traits from within the millions of accessions held in genebanks, like ‘finding the proverbial needle in a haystack’ [13]. Therefore, to improve the utilization of germplasm, genebanks are increasingly required to move beyond providing basic passport data that defines only the identity and origin of the genetic resources, to thoroughly catalogue and make publicly available additional information for accessions such as agronomic, physiological, and genetic traits that meets the specific needs of end-users [14,15,16].

McCouch et al. [17] proposed a strategic three-step approach to effectively mine genebank genetic resources that combines genomics and phenomics with efficient database management to enhance the value of available germplasm that is readily available to breeders. Although the use of genomics by genebanks have advanced due to the development of DNA technology and next-generation genome sequencing [18,19], genebank phenomics still lag in the valorizing of available plant genetic resources [20,21]. The lack of robust, cost-efficient phenotyping tools and systematic collection of phenotypic data of accessions are currently a bottleneck, restricting the exploration and utilization of genebank genetic resources for downstream research and breeding [22]. A vast amount of useful agronomic and physiological information from genebank seed regeneration trials are not systematically recorded, contributing to the underutilization of germplasm [5]. Since standard genebank characterization practices can be expensive and time-consuming, a strategic cost-effective approach for simultaneously collecting multiple phenotypic trait data from genebank accessions during routine annual seed regenerations is essential to efficiently collect this valuable data and provide it to end-users [7,23,24]. This phenotypic data can be readily available for use in combination with genotypic information in subsequent genomic studies and breeding purposes [19,25]. High-throughput phenotyping (HTP) using sensors and imagers is a promising, efficient, and cost-effective approach to collect phenotypic data for multiple traits across large scale trials, that can then be used together with genomic data for accurate selection in breeding [26,27,28]. This approach has been successfully applied for genomic selection in wheat using various sensor-derived representations of agronomic and adaptive traits [29,30].

Although there are numerous excellent reviews on genebank mining using genomic approaches [13,18,31,32], only a handful of literature has addressed the exploitation of plant genetic resources using a phenomic approach is available and even do not fully cover genebank management practice as a whole [17,33,34,35]. In this manuscript, we examine: (i) current HTP methods that can be applied to phenotype accessions to leverage genebanks’ phenotypic dataset; (ii) compatible crop traits that can be phenotyped by HTP technology and catalogued together with passport data in a genebank database; and (iii) data management strategies to effectively exploit these phenotypic data for future use. Finally, we discuss the challenges and future perspectives of genebank phenomics. Although there are numerous HTP methods, we limit our discussions to those that are more applicable to characterize and evaluate genebank germplasm in accordance with the international crop descriptors.

## 2. Phenomics to Unlock the Genetic Potential of Genebank Germplasm

### 2.1. Plant Phenomics and Its Potential Applications for Plant Genetic Resources Research

Plant phenomics is a multidisciplinary field that enables the systematic and comprehensive research and development of robust HTP tools and methods for data capture, processing, handling, and meta-analysis of phenotypic properties, growth, the performance of crops, and their environments [22,36]. The foundation of plant phenomics is the advent of HTP technology, which contrasts with more conventional arduous manual and destructive phenotyping, as it uses sensor- or image-based instruments to non-destructively simultaneously measure morphological, agronomic, and physiological characteristics of crops on a large scale across time and space. HTP technology is fundamentally based on principles of interaction between plant cellular components and natural light spectra between 400–2500 nm [37]. By capturing and analyzing these interactions proximally or remotely, important morphological, agronomic and physiological properties can be derived such as crop growth status, phenology, water and nutrient content, and yield potential [38]. The HTP approach has been widely used for decades in agriculture and plant science research with promising outcomes [39,40].

Various HTP platforms that use a combination of multiple sensors have been developed over many years that are suitable for plant science research in controlled and field conditions [27,39]. In the controlled environment, the system such as the automated Scanalyzer 3D imaging platform developed by LemnaTec GmbH (Aachen, Germany) has been effectively used to phenotype various crops and traits [41,42]. In the field, many HTP platforms are currently deployed such as the Field Scanalyzer gantry type [43]; manned- [44] or unmanned ground vehicles (UGV) [45]; and manned- [29] or unmanned aerial vehicles (UAV) [46,47]. These platforms are equipped with multiple sensor types and can be used to capture various crop traits at the same time. These sensor technologies will continue to advance over time and are likely to become less expensive, and hence more affordable for use in plant science applications.

Sensors developed for HTP can be broadly classified into either active or passive sensors that need to be considered when used to capture data. Passive sensors measure reflectance coming directly from natural light, thus the data captured by these sensors are highly affected by environmental conditions. Examples of passive sensors are Analytical Spectral Devices (ASD) FieldSpec spectroradiometer [48,49]; red-green-blue (RGB) [50,51], multi- [52] and hyper-spectral [53], and thermal cameras [54]. Active sensors, however, use their own light source and therefore the resulting reflectance is much less affected by the environment, with crop circle [42] and light detection and ranging (LiDAR) [55] being typical examples. Regardless of which type is deployed, sensors must be well calibrated and raw data should be normalized before analysis for quality assurance. Individual or multiple sensors can be handheld and mounted on vehicles or platforms, depending on the experimental setup and availability [56].

The workflow of deploying sensors for phenotyping crop experiments usually involves three main steps: 1) data capture; 2) raw data processing and storage; and 3) validation and comprehensive data analysis. Raw data are captured by sensors and processed by computer software algorithms to derive digital plant parameters such as vegetation indices (VIs) or structural properties. Once validated by compatible ground truths from conventional observations, these digital parameters can be used as proxies of crop traits for subsequent analysis. For instance, one of the most common vegetative indices is the normalized difference vegetation index (NDVI), which is derived from red and near-infrared (NIR) spectral bands and is widely used as a representation of biomass, grain yield, and crop N status [57]. The 2D and 3D structural models can be reconstructed from RGB, multispectral, and thermal imagery to derive important agronomic traits for various crops under different environments such as flowering time of rice [58] and wheat [43]; crop biomass of field peas [42] and wheat [41]; plant height and biomass of rice [59] and barley [60]; seed characteristics of lentils [61], rice [62], and field peas [63]; architectural and physiological properties of apple trees [64]; height and morphological characteristics of blueberries [65]; canopy temperature of black poplars [66]; bunch architecture of grapevines [67]; and ripeness estimation [68] and fruit counts [69] of mangos. Recent advances in computer algorithms and machine learning have significantly improved the throughput of raw data processing and analysis, where the processing pipelines have enabled data capture, analysis, and extraction of multiple patterns and features simultaneously [70]. Machine learning in sensor- and image-based phenotyping has been applied successfully for germination assessment of tomato seeds [71], head count [72,73], yield prediction in wheat [74], and prediction of seed longevity in oilseed rape from chemical compositions [75].

Since thorough discussions on the development and application of HTP tools for agriculture research alone are not the main purpose of this review; readers can find detailed information about sensors and platforms, image processing and storage, data analysis approaches from numerous excellent reviews, and references cited therein [22,26,27,36,76,77,78,79].

### 2.2. Why Genebank Phenomics?

There are multiple factors, both subjective and objective, that make genebank phenomics feasible and strategic, i.e., the availability of cost-efficient HTP technology; the nature of routine operations; the pressure to efficiently exploit genetic resources for crop improvement and the conservation of genetic diversity. Phenotyping is the most expensive yet indispensable component of any plant research and crop improvement program to understand the genetic basis and interaction between genotypes and environments. The use of HTP tools and methods discussed above by genebanks will shorten the time requirement, increase throughput, improve consistency, reduce the overall cost of phenotyping projects, and improve selection accuracy in breeding programs, especially for large-scale trials [80].

Genebanks complete essential seed regeneration as routine practices to maintain the viability, quality, and quantity of accessions, e.g., when the quantity and viability of specific accessions fall below a standard threshold [7,81]. Characterization of germplasm for a range of phenotypic traits is undertaken during the regeneration process, however, traits are manually recorded, can be subjective, and are time-consuming to collect, limiting the amount of data able to be captured, resulting in a wasted opportunity for a comprehensive characterization of genetic materials, with the flow-on effect of restricting their subsequent utilization. Mining superior agronomic alleles for breeding is crucial for improving crop yield and resilience, with the availability of comprehensive phenotypic data for genebank germplasm enabling researchers and breeders to more accurately identify desired accessions for breeding projects [82].

The application of low-cost HTP methods to assess the true value of genetic resources, accurate estimation of their agronomic phenotypic traits for a complete phenotypic representation of collections will significantly improve the gains of pre-breeding or breeding programs with marginal extra expenses. This is particularly useful for studying complex traits such as grain yield. Multiple secondary traits captured by HTP tools that correlate well with target traits (i.e., grain yield) can be used as surrogates in yield selection models to improve prediction accuracy. For instance, Rutkoski et al. [29] showed that the use of canopy temperature and NDVI measured by aerial thermal and hyperspectral sensors substantially improved genomic and pedigree yield predictions of 557 wheat lines across five growing environments. Interestingly, the authors also pointed out that genetic value for grain yield can be accurately estimated by using these secondary phenotypic traits in absence of pedigree and genomic data. The phenotypic profiling of genebanks’ accessions can, therefore, provide direct support for phenomic selection or choice of parents in breeding programs.

Genomic selection has been proven as an excellent tool to estimate genomic breeding values and is now widely used as a routine selection method in crop breeding [83,84]. However, since its successful introduction over the last two decades, there has been a significantly faster loss of genetic variance in breeding programs compared to conventional phenotypic selection [85]. To slow down the loss of genetic diversity through genomic selection in plant breeding, a physiological breeding approach combining multiple integrative traits captured by HTP tools in conjunction with genomic selection methods, with a heavier weight placed on phenotypic components, could be an alternative [86]. The advantage of phenomic selection has been demonstrated by Rutkoski et al. [87], where the authors claimed that using an optimized breeding scheme with phenotypic selection for quantitative analysis of stem rust resistance in wheat would result in equal genetic gains as genomic selection, but higher genetic variance. This phenotypic selection approach is further supported by a recent study of Rincent et al. [88], where distinctive endophenotypes, such as transcripts, small RNAs, or metabolites, could be used as phenomic markers for the selection process. The authors found that the matrices of near infrared spectroscopy absorbance between 400 and 2500 nm of winter wheat grains and leaf tissue could provide better yield prediction than molecular-based markers. Thus, using these low-cost, high-throughput endophenotypic markers significantly improved genetic gains, while better conserving allelic diversity of breeding populations.

Finally, safeguarding genetic resources ex-situ for integrity, diversity, and allelic variability for future use is the mandatory task of every genebank whose materials fuel breeding programs, underpinning food security efforts and bringing billions of dollars in benefit [17]. For instance, by 1997, the world economy had benefited approximately $115 billion annually from using wild materials from genebanks to develop environmentally resilient and resistant crops [89]. Selections for desirable agronomic traits are the driving force of plant domestication and crop improvement. However, extensive breeding selections lead to loss of genetic variants, narrowing a crop’s genetic base and an overall erosion of crop diversity through breeding programs [40,90]. Alarmingly, there is also mounting evidence that indicates that allelic variance of genebank accessions might be lost over time through seed regeneration due to genetic drift and inbreeding, while its storage size and maintenance costs will keep increasing [91]. Genebank accessions are collected from various geographical locations, thus original phenotypic variance could be lost during ex-situ conservation and seed regenerations [92]. While DNA fingerprinting is the most effective method to verify the genetic integrity of regenerated materials, the associated genotyping cost is still too high for large scale genotyping of thousands of accessions per year [62]. Thus, a complete phenotypic assessment of accessions through periodic seed regenerations could be a counter measure to ensure that original phenotypic features are preserved. Furthermore, those accessions possessing desirable agronomic characteristics can be recommended for immediate use, whereas those which do not have attributes of immediate interest can be conserved for further evaluation under different and specific environmental conditions, or potentially be discarded.

## 3. Phenomic Characterization and Evaluation of Genebank Accessions

Missing or incomplete passport, characterization, and evaluation data is one of the main reasons for the underutilization of genebank germplasm. For decades, the crop descriptor lists developed by Bioversity International have been routinely used to standardize genebank data collection and to facilitate the exchange of information between genebanks and end-users. This data is also used by genebanks to catalogue morphological and physiological characteristics of various crop species for germplasm validation processes [8]. In this section, we discuss the potential of deploying HTP technology to routinely collect quantitative data of specific traits in line with these descriptor lists and the possibilities of collecting additional data with a marginal cost that can enriches genebank collections. Highly heritable morphological and physiological features can provide invaluable information for strategic selection schemes used by plant breeders to speed up the development of new, high yielding environmentally adaptive cultivars [93]. Table 1 details systematic HTP approaches of genebank germplasm for morphological and physiological traits in different environments.

### 3.1. Morphology

The collection of data on the morphology of accessions is a critical part of routine curatorial activities of any genebank. These data describe overall plant architecture, height, leaf shape, and angle. Conventionally, these data are visually assessed and manually recorded by curators, which are sometimes subjective in nature and prone to human errors. This labor-intensive and time-consuming notetaking can be replaced by robust HTP technology (Table 1). Morphological characteristics of various crops such as number of tillers (wheat) [94]; node and internode length (tomato) [95]; panicle, branch, and leaf number (rice, maize, tomato) [97]; and leaf shape (legumes) [102] can be easily acquired by cost-effective RGB imagery tools. The HTP technology can be flexibly applied for trait capture under various growing conditions including field and greenhouse environments. For example, plant height is an important botanical trait that is defined as the shortest distance from ground level to the upper boundary of photosynthetic tissues [145]. It is a useful indicator of crop growth rate, biomass, yield potential, and lodging resistance [46, 132]. Studies on wheat have shown that lodging can cause yield losses up to 80% [146]. Thus, strategic exploitation of genebank germplasm for novel alleles is crucial for the development of lodging resistant cultivars. Several HTP methods using sensors such as ultrasonic sensors, LiDAR or RGB cameras can be used to measure plant height in the greenhouse and field [98]. However, the method using a combination of LiDAR and RGB camera mounted on a ground- or aerial based vehicle appeared more feasible with a similarly high level of accuracy [147]. Using this method, plant height can be modelled and estimated by the principles of the structure from motion photogrammetry, where the difference between digital terrain model (DTM) and digital surface model (DSM) is the average height of plants within the plots [99]. Quantitative measurement of lodging can be derived from the differences between DSM before and after lodging events, which has been demonstrated in barley [131], wheat [132], and rice [133].

### 3.2. Inflorescence and Fruit

Inflorescence and fruit are important and distinctive botanical features of crops used to identify and classify genebank accessions. In physiological breeding, highly heritable traits in cereals such as spike length, spike weight, and floret number per spike are indicators of agronomic values, yield, and adaptation for selection schemes [146]. These traits can be quantitatively measured on large-scale seed regeneration trials by using cost-effective HTP technology (Table 1). For instance, Grillo et al. [148] developed a method to differentiate wheat landraces by glume size, shape, color, and texture using a color scanner. Likewise, Makanza et al. [118] designed a simple low-cost RGB imaging method to quantitatively measure seed size, number, and weight of intact maize cobs in the field. Most recently, Genaev et al. [113] described a simple RGB imaging setup which can precisely quantify morphological features such as spike shape and awnedness of the wheat spike. This work suggests that the deployment of HTP methods can help curators digitally characterize a wide range of traits related to the inflorescence and fruit, and once incorporated into genebank databases, readily provide this quantitative data for subsequent genetic analysis and breeding purposes by end-users.

### 3.3. Seed Characteristics

Seed traits such as shape, size, and coat color are crucial criteria for determining commodity market values and are highly controlled by genetics. Genebanks routinely characterize seeds based on general morphology and use the data for both in-house quality assurance and end-user purposes. Currently, most genebanks manually collect this data following seed regeneration cycles based on visual assessment of traits, an approach that can be subjective and potentially lead to inaccurate results. Image-based phenotyping methods using RGB, multispectral, and hyperspectral cameras could be a cost-effective and accurate substitute for manual phenotyping since shape, size, and coat color are easily reconstructed and analyzed using reflectance spectra from the seed surface, and are known to be related to chemical properties (Table 1) [149]. These HTP tools have been applied for seed quality, purity, viability, vigor testing, and variety identification on various crop species [150,151]. Potentially, this cost-effective HTP technology can be used to develop seed descriptor states for crop species [120,152] and as routine methods for managing genebank accessions as they are included into the collection from new acquisitions or seed regeneration events to avoid physical contamination and maintain genetic integrity [62], as well as genomic selection for seed traits [153].

### 3.4. Phenology

Understanding the timing of key physiological growth stages such as germination, flowering, and their variations is critical for crop production and breeding of new high yielding and environmentally adaptive varieties. Therefore, the documentation of crop phenological traits such as germination, flowering, and maturity are routinely recorded during genebank seed regeneration cycles. Research has shown that image-based phenotyping can be used to effectively measure these qualitative traits as a replacement to conventionally visual-based methods. For instance, HTP technology has been used for phenotype emergence [123,124], heading, and flowering [43,129] of various crop species (Table 1). This also suggests that these trait data can be systematically and simultaneously captured together with other traits by using HTP technology, reducing the cost and increasing the opportunity to explore the genetic potential of individual accessions.

### 3.5. Physiological and Agronomic Traits

Most genebanks choose to skip the collection of physiological and agronomic traits (known as evaluation data) from their standard curatorial procedures due to either funding shortage or the labor intensity required. However, this information together with genebank passport data is critical for prioritizing and enhancing the utilization of valuable germplasm for the selection of parents used for breeding [35,154]. Core collections can be generated using phenotypic data of useful agronomic traits [155]. A plethora of reports and publications from multiple international research groups have indicated that specially designed HTP platforms can comprehensively capture multiple trait data simultaneously that can be subsequently exploited not only by curators but also by plant scientists and breeders (Table 1).

This is particularly helpful when dissecting the genetic basis of polygenic traits such as grain yield or adaptive traits. For instance, grain yield is a critically important trait for selection in physiological breeding and can be effectively captured along with other descriptive traits using HTP technology during standard curatorial procedures at genebanks. However, grain yield is a genetically complex trait that can only be improved by simultaneously enhancing other secondary morphological and physiological traits such as plant architecture, lodging resistance, photosynthetic capacity, canopy temperature, and harvest index. This approach has been proposed for major agricultural crop species such as wheat [86,156], rice [157], and pulses [158]. Therefore, the use of HTP technology has a distinctive advantage over the conventional manual collection method, where the former can capture multiple secondary trait data quantitatively at the same time. This will reduce time, labor, and phenotyping cost with the benefit of a comprehensive data set, fully describing crop growth and yield, which is critically valuable to breeding programs. Distinctive secondary traits can also be directly used to breed for adaptation or indirectly in forward genetics for molecular cloning and gene identification. For example, stay green is an adaptive trait that provides better drought tolerance and nitrogen use efficiency in crops [56,159]. Research has shown that stay-green is a part of the drought adaptation mechanism that increases yield stability and lodging resistance in sorghum and other cereals, where it can lead to prolonged grain filling duration and improve yield [146,160]. Interestingly, other reports show that canopy temperature (CT)—an indicator of evaporative cooling from the canopy surface and an adaptive trait for high yielding and drought tolerance—is associated with stay green and deeper roots [161]. Thus, a HTP approach using a combination of sensors can capture stay green and CT traits together with other traits such as NDVI, height, biomass, and ground cover, as well as being used for selection in breeding programs [162,163].

## 4. Challenges

### 4.1. Lack of Resources

Despite the enormous potential to phenotype and characterize genebank germplasm to enhance genetic gain in plant breeding, there are several constraints that genebanks must fully address before being able to move forward. The first and perhaps the biggest challenge is the availability of resources for a long-term phenotyping scheme [7]. Although HTP phenotyping of genebank germplasm will provide valuable information for end-users, the associated cost for purchasing, establishing, and operating of sensors, phenotyping platforms, analysis, validation, and making available phenotypic data in a searchable online platform, respectively, is not a trivial task, and might not be affordable by every genebank [164,165]. Hidden costs such as equipment and database maintenance, software licensing, and upgrades need to be considered. Therefore, genebank managers need to carefully consider the balance between investment, labor cost, and achievement of goals before initiating HTP projects. For instance, low-cost simple HTP tools such as PhenoBox [166] can be developed for effective phenotyping of seed regeneration in the greenhouse without the need for complicated and costly automated phenotyping platforms reported by Nguyen et al. [41,42]. More importantly, in contrast to short-term research projects, genebanks are long-term investments with large numbers of accessions that require well planned, consistent phenotyping programs to be in place. Adequate planning and resources must be made available for effective phenotyping to be undertaken over the long-term if research and breeding programs are to achieve the increased plant production required in the future.

### 4.2. Technical Difficulties in Data Management and Analysis

Data capture, standardization, quality assurance, and analysis are technical challenges related to genebank phenotyping. HTP technologies generate a large volume of ‘big data’ in a short period of time using standardized protocols. However, a high level of infrastructure investment and a multidisciplinary approach for the appropriate storage, back up, data management, and analysis is required [167,168]. These data must be thoroughly validated before they can be used. In contrast to genomic data, plant phenotypes are non-constant, plastic, and change over time, as they are the results of instantaneous interactions between genotypes and the environments [169].

Furthermore, phenotypic data of field seed regenerations, mainly collected by passive sensors and cameras, are highly influenced by spatial and temporal climatic conditions and must be processed through sophisticated computational algorithms before the data can be made readily available for genebanks scientists [26]. Therefore, if data collection is not standardized for unpredictable weather and changes in agronomical practices, over the years data analysis will become difficult due to the disparity between different data sets, rendering the HTP efforts useless [23]. To cope with fluctuating climatic conditions, Xu [170] introduced the strategic ‘envirotyping’ approach, where local environmental data such as soil, weather, biotic factors, and crop management practices are documented as metadata together with plant phenotypic data.

All data must be integrated into a well-structured and publicly searchable database for end-users [171]. For instance, the Genebank Information System (GBIS) of the Leibniz Institute of Plant Genetics and Crop Plant Research (IPK), Gatersleben, Germany, currently houses approximately 151,000 crop accessions and is comprehensively managed across passport data, seed/line management, taxonomy, phenotypic characterization, and evaluation data [172]. To ensure phenotypic data generated from HTP are fully described and annotated, the plant phenotyping community has recommended a convention on the minimum information about plant phenotyping experiments (MIAPPE), where all experimental conditions are well described and published together with phenotypic data [173]. Wilkinson et al. [174] introduced the FAIR principles (findability, accessibility, interoperability, and reusability) for the management of scholarly data, where their application will enhance the handling, sharing, reuse, and interpretation of data and metadata. In addition to passport and phenotypic data, images demonstrating morphological features, which are not easily analyzed and represented by numerical data, should be included [175]. Clearly, the systematic collection of phenotype and metadata and its stewardship will assist genebank scientists to fully describe the datasets and conditions where seed regenerations are conducted and enable the interpretation of the phenotypic plasticity in statistical models.

### 4.3. Users’ Awareness

Finally, the communication of genebank data sets to the research and breeding community is critical for increasing the successful utilization of genebank germplasm. Currently, only around 10% of genebank germplasm is used in plant breeding due to a range of technical reasons including lack of good quality phenotype and genotype data on the germplasm, as well as accession’s low level of adaptation to changing environments and genetic drift [11]. Although accurate phenotypic data are valuable to plant breeders to identify outstanding performers as potential parents, phenotyping is still the most expensive part of any breeding programs [176]. Breeders are often reluctant to take risks screening a large amount of diverse genebank germplasm without any certainty of their genetic potential for beneficial agronomic traits due to the cost and significant challenge for them to identify valuable germplasm or novel alleles from a ‘sea of seeds’ [177]. In this context, readily available phenotypic and genomic data that enriches genebank passport data will enhance the utilization and overall value of germplasm stored in genebanks.

## 5. Future Perspectives

### 5.1. Systematic HTP Phenotyping of Routine Genebank Seed Regenerations

**Figure 1:**
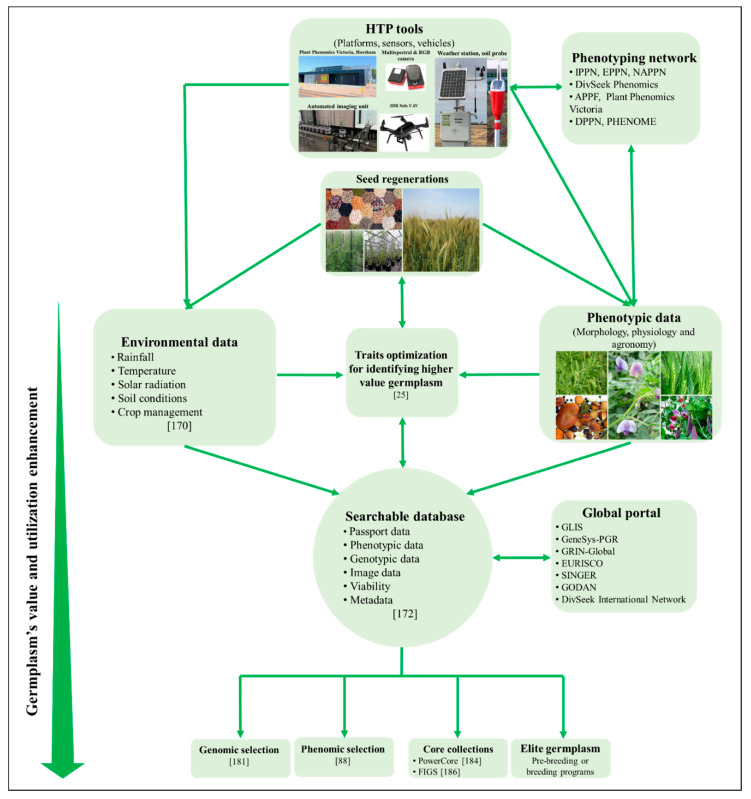
A proposed strategic phenomic approach to improve the value and utilization of genetic resources. IPPN, international plant phenotyping network; EPPN, European plant phenotyping network; NAPPN, North American plant phenotyping network; APPF, Australian plant phenotyping Facility; DPPN, German plant phenotyping network; PHENOME, French plant phenomic infrastructure; GLIS, global information system; GeneSys-PGR, global portal on crop genetic Resources; GRIN-Global, global germplasm resource information network; EURISCO, European plant genetic resources search catalogue; SINGER, system-wide information network for genetic resources; GODAN, global open data for agriculture.

Despite the challenges posed by the deployment of sensors and image-based HTP protocols, systematic collection of data from genebank seed regeneration cycles can effectively derive multiple trait data for downstream research. One of the advanced features of HTP is that multiple sensors can be deployed at the same time to simultaneously and non-destructively capture many independent observations that will allow for more targeted prioritization of accessions from large genetic resources collections for downstream studies. Targeted beneficial endophenotypes of individual genebank accessions can be directly used for the low-cost phenomic selection in breeding process or prioritize germplasm for higher value in the selection of crossing parents [88]. A proposed strategic phenomic approach for the collection of multiple trait data, management of genebank collections, and increasing utilization of data and seed by end users through the adoption of HTP technologies is shown in Figure 1.

Routine seed regeneration protocols of genebanks are often conducted in small, unreplicated plots or even single rows in the field or pots in greenhouses. Seed regeneration blocks should be replicated with a reasonable number of individuals whenever possible to facilitate statistical analysis and ensure sufficient number of seeds are used to maintain the genetic diversity and integrity of accessions (Figure 1) [178,179]. A large amount of morphological, agronomic, physiological (Table 1), and environmental data [170] can simultaneously be collected from routine seed regeneration cycles over subsequent years using HTP (Figure 1). Even though these phenotypic data are highly incomplete [23], meaningful inferences can still be achieved using appropriate analysis methods, such as identifying novel alleles [25]. Measurement of grain yield from small seed regeneration plots is generally not meaningful and is sometimes impractical to measure when thousands of lines are being regenerated in a single sowing event [30]. A practical and cost-effective phenotyping approach could be used to measure secondary correlated traits such as early vigor, height, canopy properties, and biomass during the growth phase which are components contributing to grain yield. Moreover, these phenotypic data can be instantly used in conjunction with genetic data generated by advanced genotyping technologies such as diversity array technology combined with next generation sequencing (DArT-Seq) and appropriate data quenching for direct phenomic and genomic selection from landrace accessions (Figure 1) [180,181,182].

**Figure 2:**
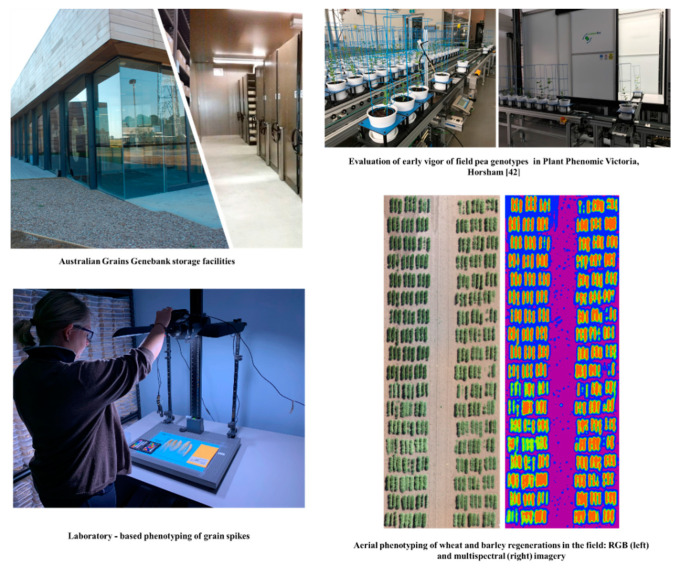
Australian grains genebank (AGG) storage facilities and its application of HTP technology for phenotyping routine seed regenerations in laboratory, greenhouse and field.

This strategic phenomic approach has been deploying at genebanks elsewhere. For example, the Australian grains genebank (AGG), Horsham, Victoria was established in 2014 and currently houses approximately 195,000 accessions of 918 crop species such as wheat, barley, canola, field pea, chickpea, lentil, sorghum, maize, cowpea, mungbean, and millets. The number of accessions has increased by around 2750 per annum (Figure 2) [9]. Annually, AGG regenerates more than 3500 accessions of genetically diverse crop species and wild relatives in the field and greenhouse, subject to viability, quantity in stock and user demand (Figure 2)[9]. This routine activity requires large inputs of labor and resources costing in excess of A$500,000 per annum. Due to the large number of accessions being regenerated annually, using conventional phenotyping methods to obtain a complete phenotypic data set is not possible. Several field HTP platforms can acquire multiple crop traits such as plant height, biomass, leaf area index, and canopy temperature across thousands of seed regeneration plots at the same time [54,99,136]. The AGG is currently applying different HTP platforms such as automated phenotyping of Plant Phenomics Victoria, Horsham [41,42], laboratory-based phenotyping of spikes and airborne platforms (Figure 2) to capture more useful morphological, agronomic, and physiological traits from seed regeneration cycles. Once validated and analyzed, these data will be made publicly available with passport data which will help end-users prioritize higher value germplasm for targeting traits in subsequent studies (Figure 1).

### 5.2. A Combination of Genebanks’ Data Mining Approaches

To enhance value and utilization of germplasm, it is crucial that traits are identified and linked with genebank accessions (Figure 1). Several methods have been proposed for mining genebank data such as using published data sources and users’ feedback, core and mini core collections. phenotyping and genotyping approaches [15]. Overall, these methods aim to identify genebank accessions containing agronomic traits of interest. Core collections can be constructed by using phenotypic (Figure 1) [88,183], genomic [13], and geographical information of accessions for certain crop traits. A variety of software packages are available to develop core collections solely using phenotypic traits, for instance, Chung et al. [184] analyzed 11 quantitative and 28 qualitative phenotypic traits from 10,368 characterized rice accessions and derived a core collection of 107 entries by using PowerCore software. Similarly, Dutta et al. [185] constructed a core set of 2,208 accessions from 22,469 accessions of wheat and its wild relatives used 34 highly heritable phenotypic traits.

Accessions can also be grouped based on the geographical information data as specified in the focused identification of germplasm strategies approach (FIGS) [186,187]. The underlying principle of the FIGS approach is that crops will likely evolve under environmental selection pressures and develop their adaptation in response to extreme climatic conditions. Thus, the method uses detailed eco-geographical location and weather conditions where accessions were collected to precisely predict their adaptive traits to abiotic and biotic conditions. The FIGS approach has been successfully used to identify several core sets such wheat stem rust [188], drought in faba bean [187], powdery mildew in wheat [189], and Russian wheat aphids [190]. Using these data mining approaches will narrow down the number of accessions for further analysis while the allelic variance is still well maintained in the subsets.

Several approaches can also be used in sequence to increase the chance of identifying targeted accessions as reported by Haupt and Schmid [191] where two core collections of 183 and 366 soybean accessions were chosen from the original collection of more than 17,000 accessions by using a combination of FIGS approach and SNP genotypic markers. Given the advanced features of HTP, capturing multiple traits nondestructively at the same time, core collections can be easily developed and accessions containing promising traits of interest will be chosen for further studies.

### 5.3. A Collaborative Network for Data Collection, Analyzing and Sharing

To improve HTP practices, genebanks should work closely with universities, research institutes and industries to standardize seed regeneration procedures, phenotyping protocols, and calibration of sensors so that the resulting phenotypic data are comparable across genebanks and able to be fully exploited by end-users. Numerous initiatives have been implemented at the national and international scale, which aim to bring academia and industry together to address common phenotyping questions and integrate the plant phenotyping community (Figure 1) [171]. For instance, world-class plant phenotyping infrastructures have been established in Australia to enhance the capability, capacity, and scientific rigor in support of national plant phenomics studies and applications. These include the Australian plant phenotyping facility with three nodes located at the University of Adelaide, the Australian National University, and the Commonwealth Scientific and Industrial Research Organization, Canberra, respectively. Moreover, the Plant Phenomics Victoria is home to two nodes located in Horsham and Bundoora, Victoria (Figure 1). At the global scale, the International Plant Phenotyping Network [171] is an organization representing plant phenotyping centers, that formulates multidisciplinary working groups and enables the communication between stakeholders through conferences and training workshops so that up to date information about new HTP infrastructures and methodologies for various crop phenotypes can be effectively shared (Figure 1). This networking collaboration is essential to foster the advancement in plant phenotyping technologies including affordable phenotyping, sensors, and platforms, targeting traits and data analysis pipelines and data management.

To make valuable information pertaining to germplasm available for global users, a cooperative platform for data collection, analysis and sharing is urgently required. Several international initiatives such as DivSeek, breeding API, research data alliance (RDA), and global open data for agriculture (GODAN) have been developed, all of which have aimed to facilitate the integration and sharing of evaluation and characterization data so as to improve the value and utilization of germplasm [192]. For instance, the DivSeek international network is a global, community-driven initiative that facilitates the cooperation and interactions among its members through working groups [17]. Genebanks, phenotyping scientists, and breeders can develop and share methodologies, tools, and best practice phenotyping technologies to evaluate genetic resources, which improve the generation, integration, and sharing of phenotypic data [193]. Moreover, the introduction of the global information platforms such as the global portal on crop genetic resources (GeneSys-PGR) has enabled breeders and genebank users to use free online search engines to explore and request germplasm accessions conserved in genebanks worldwide [177]. The global information system is an international portal that links all current plant genetic resources systems by using unique digital object identifiers (DOIs) for individual accessions [192]. By using DOIs and linkage through these portals, invaluable phenotypic evaluation and characterization of germplasm can be effectively shared with the global user network.

Individual institutions can setup different collaboration protocols for sharing and exchanging phenotypic and genotypic data (Figure 1) [172]. For example, the International Maize and Wheat Improvement Center (CIMMYT), El Batán, State of Mexico, Mexico distributes seed all over the world and receive data in return from experimental trials that provide valuable information to assess genotype-by-environment interaction [194]. A more similar stringent protocol can be introduced to enforce the current clause in the standard material transfer agreement of the seed distribution of any genebanks, where the end-users are obliged to give back basic phenotypic data of genetic resources which they have used such as trial location, phenology, biomass, and grain yield. This information would clearly enrich the genebanks’ databases and the GeneSys-PGR, which can be used as reference guides by end-users for future use. However, phenotypic and genotypic data should be linked and shared with other national and international databases of plant genetic resources through the use of DOIs and the global portals discussed above.

Although genebank scientists make use of invaluable knowledge and techniques from other research disciplines such as plant physiologist, breeders, agronomists, seed physiologists, and computer scientists, their independent translational studies are indispensable to fully utilize HTP technology to phenotype genebanks’ accessions [34]. For instance, HTP methods can theoretically be applied to quantitatively analyze the 3D canopy structure of wheat by multi-view stereo and structure from motion algorithms [50]. This protocol is not yet ready for large-scale phenotyping of genebanks accessions as translational research must be conducted by genebanks to optimize the existing protocol and determine if its throughput is applicable for large-scale phenotyping of various crop species. Similarly, more studies should be dedicated to verifying and developing feasible seed testing methods by using multi- and hyper-spectral imagery for handling genebank accessions of various crop species [62].

## 6. Conclusions

The application of HTP technology for large-scale phenotypic characterization and validation of genebank germplasm is essential if they are to fulfill their biorepository role (i.e., in the preservation and support of further experimentation and plant breeding) [195]. With a comprehensive phenomics approach combining pedigree, genomic, and phenotyping data [17], the true value of genebank genetic resources is evident. Therefore, they should more strategically and efficiently utilized by breeding programs that should double our current rate of genetic gain to feed the growing world population under the changing conditions expected into the future.

## Figures and Tables

**Table 1:** Examples of phenotypic traits can be exploited from genebank germplasm by using sensors and phenotyping platforms.

Traits	Description	Sensors and Capture mode	Species	Environment	References
Morphology					
Plant architecture	Number of tillers of wheat plants	Automated RGB^1^ imaging platform, LemnaTec 3D Scanalyzer	*Triticum aestivum*	Greenhouse	[94]
	Node and internode length of tomato seedlings	RGB imagery	*Solanum lycopersicum*	Greenhouse	[95]
	Characterization of plant architecture by 3D scanning reconstruction	Blue-laser scanner	*Solanum lycopersicum,* *Nicotiana benthamiana,* *Sorghum bicolor*	Greenhouse	[96]
	Canopy structure (tiller and leaf number, leaf length and angle, leaf elongation rate)	RGB imagery	*Triticum aestivum*	Greenhouse	[50]
	A phenotyping platform, PANorama, measuring architectural properties (panicle, branch, leaf) of various crop species.	RGB imaging unit	*Oryza sativa* *Zea mays* *Solanum lycopersicum*	Laboratory	[97]
Plant height	Sorghum plant height estimates	Ultrasonic, LiDAR^2^-Lite, Kinect camera, imaging array, UAV^3^ RGB imagery	*Sorghum bicolor*	Field	[98]
	Wheat plant height estimation	UGV^4^ and UAV RGB imagery	*Triticum aestivum*	Field	[51,99]
	Rice plant height estimation	UAV RGB imagery	*Oryza sativa*	Field	[59]
	Barley plant height measurement	UAV RGB imagery	*Hordeum vulgare*	Field	[60]
	Maize plant height estimates	UAV RGB imagery	*Zea mays*	Field	[100,101]
Leaf properties	Characterization of local leaf vein in legume leaves	Color scanner	*Vigna angularis* *Phaseolus vulgaris* *Glycine max*	Laboratory	[102]
	Leaf morphological properties and height of maize, measured by various digital phenotyping methods	3D scanner, Multi-view stereo cameras, FastTrack 3D digitizer	*Zea mays*	Laboratory	[103]
Inflorescence and fruit					
	An automated imaging system for monitoring the growths of maize ear and silks	RGB imaging platform	*Zea mays*	Greenhouse	[104]
	Analysis of panicle architecture and spikelet numbers in rice by an imaging tool, P-TRAP^5^	RGB imagery	*Oryza sativa*	Field, laboratory	[105,106]
	Rice panicle phenotyping using Panicle-SEG^6^ algorithm	RGB imagery	*Oryza sativa*	Field	[107]
	Characterization of maize tassel traits by RGB imaging and machine vision	RGB imaging sensors	*Zea mays*	Field	[108,109]
	Analysis of oat panicle development	RGB imaging platform	*Avena sativa*	Greenhouse	[110]
	Fruit recognition and counting by an imaging robot, SPYSEE	RGB imaging robot	*Capsicum annuum*	Greenhouse	[111]
	Morphological characterization of wheat spike (grain number, size and angle, stem node)	Computed Tomography imagery	*Triticum aestivum*	Laboratory	[112]
	Automatic quantification of wheat heads	Automated RGB imagery platform, Field Scanalyzer	*Triticum aestivum*	Field	[73]
	Morphometric properties of wheat spikes	RGB imagery	*Triticum aestivum*	Laboratory	[113]
Seed characteristics					
	Seed quality of field pea (color, shape, and size) analyzed by multi-spectral imaging	Built-in multi-spectral camera, EyeFoss	*Pisum sativum*	Laboratory	[114]
	Evaluating of lentil seed size by multi-spectral imaging	Built-in multi-spectral camera, EyeFoss	*Lens culinaris*	Laboratory	[61]
	Screening method to evaluate seed properties	Nuclear magnetic resonance	*Avena spp.*	Laboratory	[115]
	Rice seed shape analyzed by an image processing pipeline	Color scanner	*Oryza sativa*	Laboratory	[116]
	Analysis of maize ear, cob and kernel properties	Color scanner	*Zea mays*	Laboratory	[117]
	Estimation of ear characteristics and kernel weight in maize	RGB imagery	*Zea mays*	Field	[118]
	Morphological characteristics of wheat kernels	Color scanner and RGB imagery	*Triticum aestivum*	Laboratory	[119]
	Phenotypic classification of rice seed accessions	Multispectral imagery, VideometerLab	*Oryza sativa*	Laboratory	[62]
	Shape description of pili seed by imaging technology	Multispectral imagery, VideometerLab	*Canarium ovatum*	Laboratory	[120]
	Automated morphological characterization of rapeseed and barley seeds	Automated RGB imaging unit, phenoSeeder	*Brassica napus* *Hordeum vulgare*	Laboratory	[121]
	Automated phenotyping of oat seed properties	NIR spectroscopy, Single-Seed Analyzer	*Avena sativa*	Laboratory	[122]
Phenology					
Emergence count	Rice seedling counts	High resolution UAV RGB imagery	*Oryza sativa*	Field	[123]
	Cotton seedling detection and count	Ground-based video recording	*Gossypium hirsutum*	Field	[124]
	Germination rate estimation in tomato by color imagery	RGB imagery	*Solanum lycopersicum*	Laboratory	[71]
	Determination of plant density at emergence in wheat	High resolution UAV RGB imagery	*Triticum aestivum*	Field	[125]
Ground cover	Ground cover estimates	High resolution UAV RGB imagery	*Sorghum bicolor* *Gossypium hirsutum* *Saccharum spp.*	Field	[126]
Flowering	Automated observations of wheat flowering	CCD^7^ digital camera	*Triticum aestivum*	Field	[127]
	Heading and flowering detection in wheat	Automated RGB imaging platform	*Triticum aestivum*	Field	[43]
	Automated flowering observation in rice from a time-series RGB images	Automated RGB imaging platform	*Oryza sativa*	Field	[58,128]
	Estimation of flowering time in maize	UAV RGB imagery	*Zea mays*	Field	[129]
Physiology					
Early vigor	Early vigor of field pea seedlings	Automated RGB imaging platform, LemnaTec 3D Scanalyzer and handheld active sensor, crop circle	*Pisum sativum*	Greenhouse, field	[42]
	Wheat vigor and canopy height quantification	UGV and UAV RGB imagery	*Triticum aestivum*	Field	[51]
	Monitoring plant and canopy growth dynamics	RGB or multispectral imagery, D3P^8^	*Triticum aestivum*	Greenhouse	[130]
Lodging	Lodging score estimation in barley by aerial imagery	High resolution UAV RGB imagery	*Hordeum vulgare*	Field	[131]
	Estimation of crop lodging in wheat by aerial imagery	High resolution UAV RGB imagery	*Triticum aestivum*	Field	[132]
	Rice lodging scores estimated by UNet model derived from aerial imagery	High resolution UAV RGB and multispectral imagery	*Oryza sativa*	Field	[133]
Photosynthesis and respiration	Photosynthetic capacities in tobacco	Handheld hyperspectral sensor, FieldSpec.	*Nicotiana tabacum*	Field	[134]
	Leaf photosynthesis in maize	Handheld hyperspectral sensor, FieldSpec.	*Zea mays*	Field	[135]
	Leaf photosynthesis and relevant physiological parameters in wheat	Handheld hyperspectral sensor, FieldSpec.	*Triticum aestivum*	Field	[48]
Water soluble carbohydrates	Estimates of stem water soluble carbohydrates at different growth stages in wheat	Handheld hyperspectral sensor, FieldSpec.	*Triticum aestivum*	Field	[49]
	Predicting the quality of ryegrass (sugar)	Hyperspectral imaging platform	*Lolium perenne*	Field	[53]
Canopy temperature	Wheat canopy temperature measurement	Airborne thermography and wireless infra-red thermometers	*Triticum aestivum*	Field	[54]
	Maize canopy temperature measurement	UAV thermal and RGB imagery	*Zea mays*	Field	[136]
	Canopy temperature and vegetation indices of wheat	Airborne thermal and hyperspectral imagery	*Triticum aestivum*	Field	[29]
Stay green	Stay-green associates with low water-soluble carbohydrates in oat	Handheld active sensor GreenSeeker	*Avena sativa*	Field	[137]
	Characterization of maize green leaf area dynamics	UAV multispectral imagery	*Zea mays*	Field	[138]
	Senescence rate in wheat	UAV multispectral imagery	*Triticum aestivum*	Field	[52]
Biomass and yield	Biomass, ground cover and canopy height estimates	UGV LiDAR	*Triticum aestivum*	Field	[55,139]
	Estimation of shoot biomass by color imagery	RGB imaging platform, LemnaTec 3D Scanalyzer	*Triticum aestivum*	Greenhouse	[140]
	Grain yield prediction by canopy hyperspectral reflectance	Airborne hyperspectral imagery	*Triticum aestivum*	Field	[30,141]
	Wheat ear counts	Handheld thermal imagery	*Triticum aestivum*	Field	[142]
	Head counts in sorghum	High resolution UAV RGB imagery	*Sorghum bicolor*	Field	[143]
	Counting of wheat spikes	Handheld and UGV RGB imagery	*Triticum aestivum*	Field	[72,144]
	Wheat biomass and yield, nitrogen related traits	Automated RGB imaging platform, LemnaTec 3D Scanalyzer	*Triticum aestivum*	Greenhouse	[41]

^1^ RGB, red green blue; ^2^ LiDAR, Light Detection and Ranging; ^3^ UAV, Unmanned Aerial Vehicle; ^4^ UGV, Unmanned Ground Vehicle; ^5^ P-TRAP, Panicle TRAit Phenotyping; ^6^ Panicle-SEG, Panicle segmentation algorithm; ^7^ CCD, charge-coupled device; ^8^ D3P, Digital Plant Phenotyping Platform.
